# Sudden Unexpected Deaths and Vaccinations during the First Two Years of Life in Italy: A Case Series Study

**DOI:** 10.1371/journal.pone.0016363

**Published:** 2011-01-26

**Authors:** Giuseppe Traversa, Stefania Spila-Alegiani, Clara Bianchi, Marta Ciofi degli Atti, Luisa Frova, Marco Massari, Roberto Raschetti, Stefania Salmaso, Gianpaolo Scalia Tomba, and the Hera Study Group

**Affiliations:** 1 National Centre for Epidemiology, Surveillance and Health Promotion, National Institute of Health, Rome, Italy; 2 Medical Direction, The Bambino Gesù Children's Hospital, Rome, Italy; 3 Italian National Institute of Statistics (ISTAT), Rome, Italy; 4 Department of Mathematics, University of Rome Tor Vergata, Rome, Italy; Fred Hutchinson Cancer Research Center, United States of America

## Abstract

**Background:**

The signal of an association between vaccination in the second year of life with a hexavalent vaccine and sudden unexpected deaths (SUD) in the two days following vaccination was reported in Germany in 2003. A study to establish whether the immunisation with hexavalent vaccines increased the short term risk of SUD in infants was conducted in Italy.

**Methodology/Principal Findings:**

The reference population comprises around 3 million infants vaccinated in Italy in the study period 1999–2004 (1.5 million received hexavalent vaccines). Events of SUD in infants aged 1–23 months were identified through the death certificates. Vaccination history was retrieved from immunisation registries. Association between immunisation and death was assessed adopting a case series design focusing on the risk periods 0–1, 0–7, and 0–14 days after immunisation. Among the 604 infants who died of SUD, 244 (40%) had received at least one vaccination. Four deaths occurred within two days from vaccination with the hexavalent vaccines (RR = 1.5; 95% CI 0.6 to 4.2). The RRs for the risk periods 0–7 and 0–14 were 2.0 (95% CI 1.2 to 3.5) and 1.5 (95% CI 0.9 to 2.4). The increased risk was limited to the first dose (RR = 2.2; 95% CI 1.1 to 4.4), whereas no increase was observed for the second and third doses combined.

**Conclusions:**

The RRs of SUD for any vaccines and any risk periods, even when greater than 1, were almost an order of magnitude lower than the estimates in Germany. The limited increase in RRs found in Italy appears confined to the first dose and may be partly explained by a residual uncontrolled confounding effect of age.

## Introduction

Most deaths occurring during the first two years of life are attributable to defined causes, mainly represented by congenital malformations, malignancies, and accidents. The events in otherwise apparently healthy subjects, without any evident cause for the death, are classified as SIDS (Sudden Infant Death Syndrome) during the first year of life and SUD (Sudden Unexpected Deaths) at older ages.

During the first years of life, children receive several immunisations and some events of SIDS-SUD (in the following SUD) may occur in temporal association with vaccination. The association between vaccinations and SUD has been frequently investigated, and most of the epidemiological studies rejected any causal relationship suggested by spontaneous reports [1]–[12].

In 2003 the suspicion of a possible association between the immunisation with a hexavalent vaccine (Hexavac) and the occurrence of SUD was raised in Germany [13]. The signal was based on the observation of three deaths occurring between November 2000 and June 2003 in toddlers in their second year of life within 48 hours following the administration of the fourth dose. No signal was detected for vaccinations administered during the first year of life.

Two hexavalent products were licensed in the European Union through a centralised licensure procedure in the year 2000: Hexavac (by Aventis Pasteur MSD) and Infanrix hexa (by Glaxo SmithKline Biologicals). These combined vaccines contained antigens to immunise against diphtheria, tetanus, pertussis, poliomyelitis, hepatitis B and haemophilus influenzae type b. Since licensure the two vaccines were extensively used in some EU countries (e.g., Germany, Italy, Austria).

Italy represented the second largest market of Hexavac in the world and no signal of a possible association with the occurrence of SUD emerged from the Italian pharmacovigilance reporting system. Childhood vaccination schedules differed slightly between Germany and Italy as four administrations were recommended in Germany (at the 2^nd^, 3^rd^, 4^th^, and between the 11^th^ and 15^th^ months of life) and three in Italy (at the 3^rd^, 5^th^, and 11^th^-12^th^ months of life). Despite these differences, it was deemed necessary to carry out further investigations and a study protocol was agreed with the CHMP-EMA (Committee for Medicinal Products for Human Use – European Medicines Agency). The study was carried out on the entire Italian population of newborns over a five year period (1999–2003) and a report was presented in 2005 at the CHMP-EMA.

Since the largest use of hexavalent products was reached in 2004 we subsequently decided, with the intent to increase the power of the study, to extend the observation period and to include all infants immunised in 2004. As for the preliminary study, the main aim was to assess whether immunisation with hexavalent vaccines in the first two years of life was associated with higher short term risk of unexplained death in the Italian setting. We are now reporting the final findings of the study for the entire period 1999–2004.

## Methods

### Ethics Statement

The study protocol was approved by the Ethical Committee of the National Institute of Health with the recommendation of avoiding direct contacts with the families of the study subjects. The study was conducted according to the Italian law on confidentiality.

### Population

Around 530,000 children are born each year in Italy out of a population of approximately 57 million inhabitants. In 2001 the infant mortality rate was 4.7 per 1,000 newborn; the mortality rate in second year of life was 0.3 per 1,000 infants. As in most developed countries, mortality rates decreased during the study period (1999–2004), as part of a long term trend [14].

The reference population was the entire population of children who were resident in Italy. The study population consisted of all children who died of SUD between 31 and 729 days of age in the years 1999–2004.

### Study design

Given the universal offer of infant immunisation in Italy, the entire population was expected to be vaccinated and the limited proportion of unvaccinated subjects might not be comparable to the immunised population. To avoid this limitation, a study design only based on case-subjects, according to the case-series methodology proposed by Farrington, was adopted [15]–[19]. The entire information was provided by cases and each subject also acted as his/her own control: the observation period was arbitrarily divided into pre-defined risk period (the days immediately following the vaccination) and control period (the remaining observation period). Each event was classified as exposed if it occurred during the risk period and not-exposed if it occurred in the control period. Incidence rates in the risk period were compared with the incidence rate in the control period to obtain an estimate of the Rate Ratio (RR). Case series design specifically applies to the investigation of the association between exposures that carry a transient effect and the occurrence of acute events. In these situations the information derived by cases may provide an unbiased estimate of the rate ratios of a hypothetical cohort study conducted in the same source population.

### Identification of cases

For each death occurring in Italy a death certificate has to be filled in by a physician. Death certificates are collected at national level by ISTAT (the Italian National Institute of Statistics). For the present study, the ISTAT national database was used to retrieve death certificates of infants aged 31–729 days from 1 January 1999 to 31 December 2004.

Certificates reporting one of the following causes of death (classified according to the Ninth Revision of International Classification of Diseases - ICD IX) were selected to identify the study subjects: SIDS (ICD IX: 7980); symptoms, signs, and ill-defined conditions (other than SIDS) (ICD IX: 780-797, and 799) without mention of concomitant conditions (e.g., septic shock, congenital malformations, accident); cardiac arrest (ICD IX: 4275) without mention of congenital malformation; and foreign body in larynx (ICD IX: 9331) without identification of the foreign body causing the asphyxia or suffocation, or of concomitant conditions (e.g., congenital diseases).

All individual records of death, with the text description of the causes and of concomitant diseases or conditions, were reviewed. Potential errors in the selection of the main cause of death and/or in the coding process were identified. The outcome of the review process was either to confirm the inclusion or to exclude the subject when an identified cause of death had been reported. Moreover, the review allowed the inclusion of additional subjects when the cause of death was judged to be compatible with SUD (even though the ICD-IX code was different). For instance, we included subjects whose cause of death mentioned “sudden death” either alone or in combination with other ill-defined causes such as “cardiac arrest” or “asphyxia”. The process was carried out by the authors (GT, SSA, LPC, and MCDA) and in case of doubt a consensus was reached on the basis of collective discussion. The review process, even though not anticipated in the study protocol, was agreed with the Scientific Committee of the study. Both inclusion and exclusion decisions were adopted before collecting individual vaccination histories.

The following demographic characteristics were abstracted from the death certificates: sex, date of birth, date of death, mothers' citizenship, municipality of residence. Confirmation by autopsy was not required for the eligibility of study subjects since the results of autopsy even when requested, were not included in the death certificate.

### Vaccination histories

In Italy, immunisations included in the national program are offered free of charge and delivered by Local Health Units (LHUs). Different commercial products may be in use at the same time within the National Health Service (NHS). National vaccination surveys indicated that around 95% of newborns complete the recommended vaccination course by the end of the second year, and that around 95% of the vaccinations are administered in the LHUs [20]. Parents may also ask the family paediatrician to administer the vaccine (which in this case is paid out of pocket by parents). In these occasions, family paediatricians are required to communicate with the LHUs to keep the vaccination records updated.

The Italian infant immunisation schedule includes three doses of vaccines against diphtheria, tetanus, pertussis, poliomyelitis, hepatitis B and haemophilus influenzae type b, to be administered in the first year of life (at the 3^rd^, 5^th^, and 11^th^-12^th^ months of age); the first MMR vaccination is recommended between the 12^th^ and 15^th^ months. During the six year study period, combined vaccines have largely replaced the use of single antigens concomitantly administered. Hexavalent vaccines reached 96% of the doses administered in the first year of life in 2004 [21].

For each case the immunisation history was requested to the competent LHU. The following data were obtained: date(s) of immunisation, product(s), and batch number.

### Statistical analyses

The observation period (31–729 days) was divided into periods at risk (the days immediately following the vaccination) and control period (the remaining observation period until subsequent vaccination or death). According to the German signal and the acute onset hypothesis, three different risk periods were considered in the analyses: the day of vaccination and the following day (0–1 days); up to 7 days following vaccination (0–7 days); up to the end of the second week following the vaccination (0–14 days). For the calculation of person time, the day of vaccine administration (index day) was considered as an entire day of exposure. Risks of SUD in each of the three periods following vaccination were compared with those in the control period.

RRs were estimated for the following categories of exposure: Hexavalent products (Hexavac; Infanrix hexa); Other concomitant administration of six antigens (e.g., pentavalent + monovalent vaccines); Other vaccine administration (any other administration of antigens: e.g., pentavalent vaccines; tetravalent + monovalent vaccines; tetravalent vaccines; MMR).

Since each case acted as his/her own control, the case-series method inherently took into account confounding factors that did not vary with time over the observation period (such as variables related to genetics, socio-economic status, gender, individual frailty, presence of underlying diseases). To take into account time-dependent confounding variables, such as age in the present study, the observation period of each subject was subdivided not only by risk categories (i.e., risk and control periods) but also by age classes. RR estimates were adjusted for the following age groups: 31–80; 81–100; 101–120; 121–180; 181–360; 361–729 days.

For estimating the association between vaccination and SUD two different analyses were carried out.

**Figure 1:**
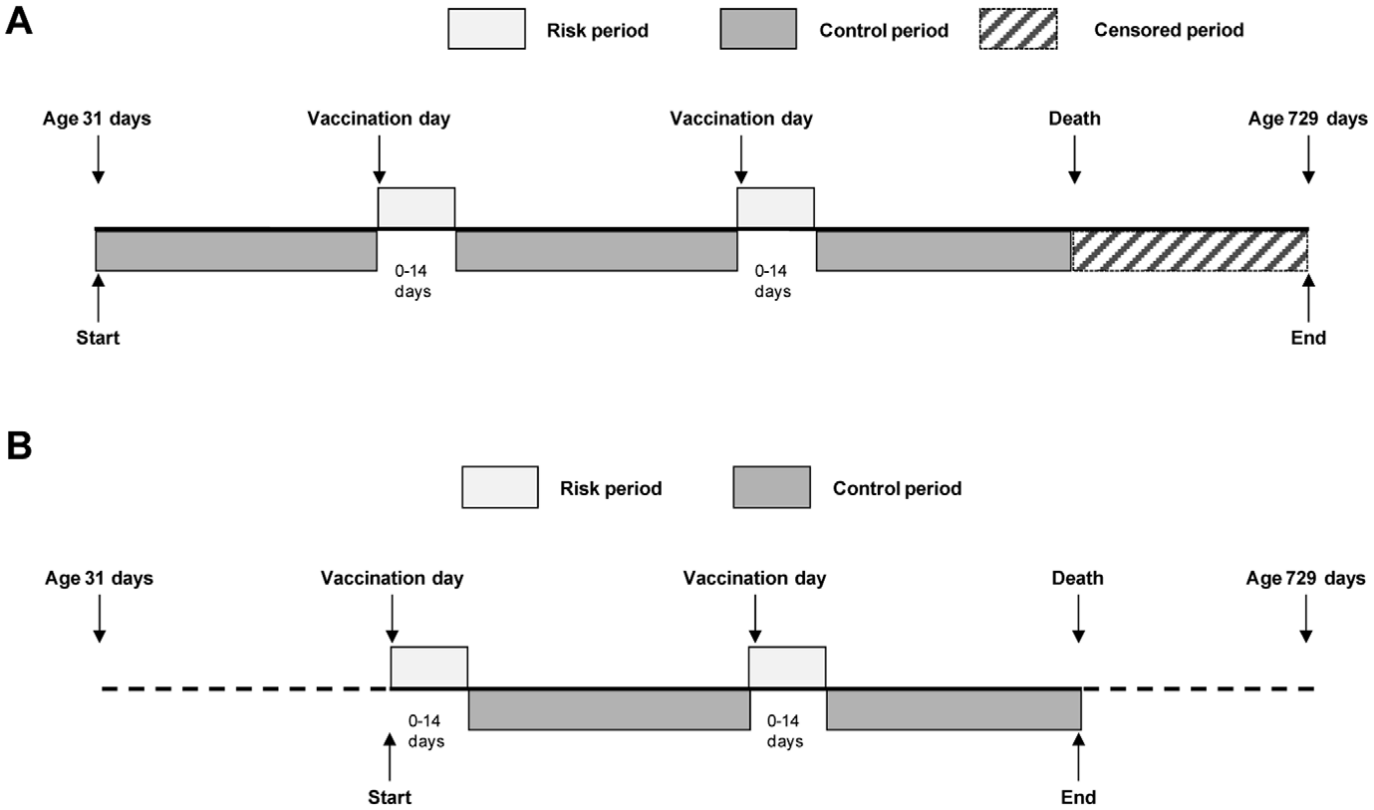
Description of the observation period for a hypothetical subject included in the study. Legend A. Self-controlled case-series method for censoring, perturbed or curtailed post-event exposures [19] Legend B. Poisson regression model.

The first analysis was based on the method developed by Farrington for self-controlled case series data (SCCS). Originally designed to analyse the association between vaccinations and recurrent events [15]–[18], the method was adapted to take into account also terminal events, as deaths, where the observation period is truncated (case series for censored, perturbed or curtailed post-event exposures) [19]. All cases of SUD (vaccinated and unvaccinated) were included in the analysis. The observation period for each case lasted between 31 days of age and the end of the observation period (729 days) (Figure 1A). RRs and 95% Confidence Intervals (95% CIs) were estimated applying a specific routine developed for the analysis of SCCS for censoring, perturbed or curtailed post-event exposures [22]. The SCCS method provides an overall estimate of the effect of vaccination (including the effect of different doses), whereas limitations are present in trying to estimate the RR of specific vaccines when different products are administered to the same child.

The second analysis was carried out to provide risk estimates for each vaccine (and categories of vaccines), taking into account that children could be exposed to different types of products. Differently from the previous analysis, only immunised children were included and each case contributed to the observation period from the day of the first vaccination to death (Figure 1B). The association between specific vaccinations and deaths was investigated using the Poisson regression model. The effect of age, sex, citizenship, and calendar year was considered. In the final model, only age, grouped in the same classes previously indicated, was included since the other variables did not modify the estimates.

Both analyses were performed with Stata Statistical Software, release 10 [23].

### Organisation of the study

The study was coordinated by the National Centre for Epidemiology, Surveillance and Health Promotion of the Italian National Institute of Health, in collaboration with ISTAT (Italian National Institute of Statistics) and Regional Health Authorities. A Scientific Committee was appointed to supervise the overall activity.

## Results

**Figure 2:**
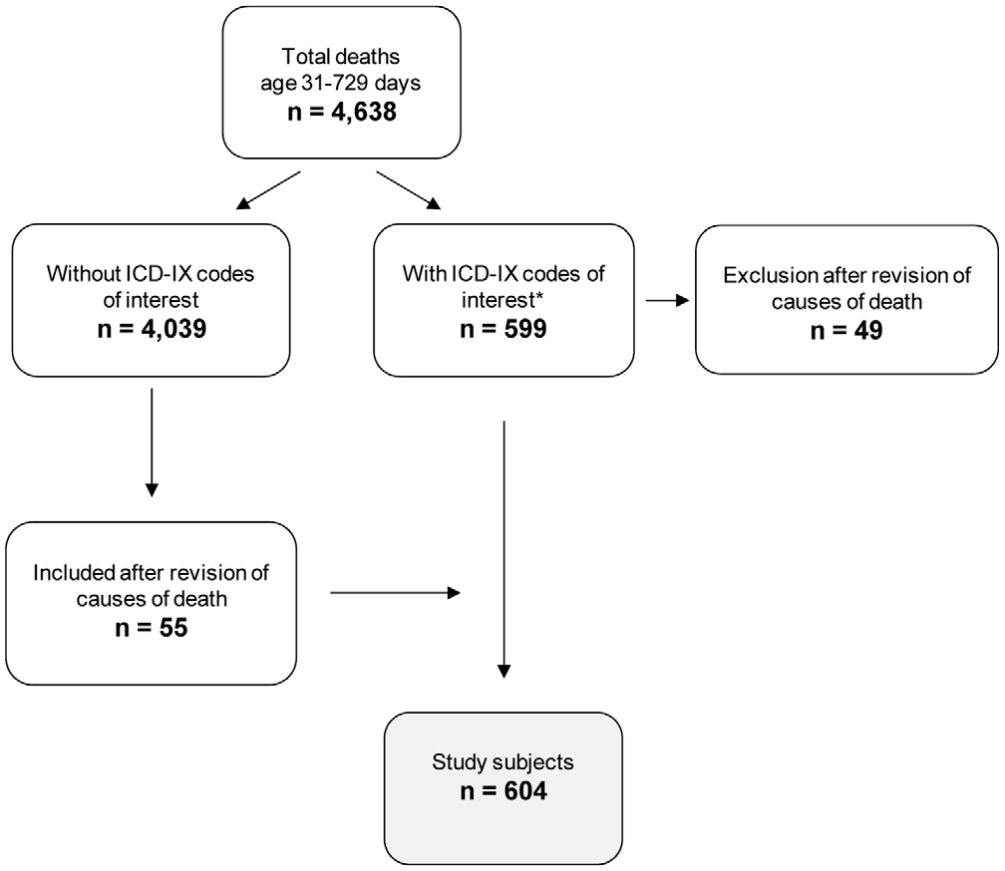
Selection of the study population, age 31–729 days, Italy 1999–2004. Legend. *One subject was excluded because the date of birth was unknown.

During the six years of the study period, 4,638 deaths occurred in Italy between 31 and 729 days of age. The ICD-IX codes of interest were reported in the death certificates of 599 subjects (one subject was subsequently excluded because the date of birth was unknown). After reviewing the description of all individual causes of death, 49 subjects originally classified with the ICD-IX codes of interest were excluded (e.g., as a consequence of concomitant reporting of septic shock or accident), whereas 55 subjects originally classified with other ICD-IX codes were added (Figure 2). The study population thus comprised 604 subjects whose death certificate was compatible with a diagnosis of SUD. The proportion of subjects included in the study (12.8%) was stable in each of the six years (range 11.3%–14.3%).

**Figure 3:**
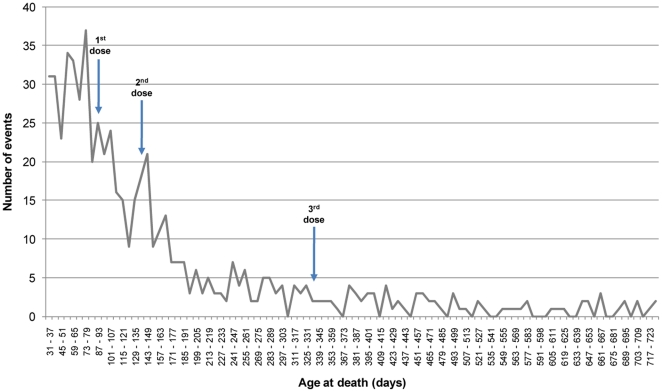
Distribution of the 604 events of SUD included in the study by age of death. Legend. The three arrows indicate the median age at first, second and third vaccine dose.

The age distribution of the 604 events of SUD included in the study clearly indicates the strong decrease in the occurrence of SUD with age, with a decline that is particularly pronounced around the age between the first and second vaccine dose (Figure 3).

The median age at death of the identified subjects was 107 days, with a greater proportion of males (male to female ratio: 1.5). The most frequently reported cause of death was “SIDS” (25%), followed by “cardiac arrest” (21%), and by “Foreign body in larynx” (11%) (Table 1).

**Table 1:** Characteristics of the study population and immunisation status.

	All subjects (N. 604)	Immunised subjects (N. 244)	
	N	N	%
**Age at death (days)**			
31–80	218	11	5.0
81–100	65	22	33.8
101–120	52	22	42.3
121–180	109	69	63.3
181–360	93	69	74.2
361–729	67	51	76.1
**Sex**			
Male	360	137	38.1
Female	244	107	43.9
**Mother's citizenship** 1			
Italian	488	206	42.2
Non-Italian	109	34	31.2
**Cause of death**			
SIDS	151	52	34.4
Cardiac arrest	124	55	44.4
Foreign body in larynx	68	23	33.8
Symptoms, signs, and ill-defined conditions (with exclusion of SIDS)	212	95	44.8
Others^2^	49	19	38.8
**Year of death**			
1999–2000	241	84	34.9
2001–2002	194	79	40.7
2003–2004	169	81	47.9
**Regional area of residence**			
North	252	122	48.4
Centre	88	23	26.1
South and Islands	264	99	37.5

SIDS: Sudden Infant Death Syndrome.

1 The information on citizenship was missing for 7 infants.

2 Causes of death judged to be compatible with sudden unexpected death after the revision of death certificates.

Out of the 604 infants, 244 (40%) had at least one recorded vaccination (Table 1). The likelihood of vaccination increased with age, from 5.0% under 80 days to 75.0% over 180 days. Non-Italian citizens and children living in southern Italy had a significantly lower probability of having the vaccination record retrieved. Almost half of the vaccinated infants (48.4%) had received only one vaccine dose, 34.4% two doses, and the remaining infants received at least three doses. The median age at each vaccine dose was consistent with the Italian immunisation schedule (Table 2).

**Table 2:** Distribution of the 244 immunised subjects by vaccine dose.

Vaccine dose	Number of subjects	%	Median age at last dose (days)	Median age at death (days)
1	118	48.4	90	125
2	84	34.4	139	220
3	31	12.7	338	422
3+MMR	11	4.5	479	552
**Total**	**244**	**100.0**	**129**	**178**

MMR: measles-mumps-rubella vaccine.

**Figure 4:**
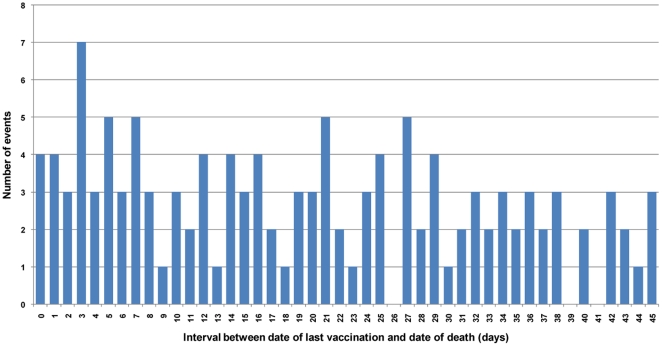
Distribution of immunised subjects by interval between date of vaccination and date of death. * Legend. *Only events occurring within 45 days from vaccination are shown.

Figure 4 reports the distribution of immunised subjects by interval between date of vaccination and date of death (during the 45 days following vaccination). Overall 8 children died on the same day of vaccination or during the following day. The age adjusted RR, estimated adopting the SCCS method for truncated observation periods (Table 3), was 1.2; 95% CI 0.4 to 2.1 (if not otherwise specified all RRs presented in the text are adjusted by age). RR for any vaccination in the time window 0–7 days was 1.3 (95% CI 0.9 to 1.9) and for the 0–14 days was 1.1 (95% CI 0.8 to 1.5). In the analysis by dose, a statistically significant RR was reached for the first dose in the risk period 0–7 days following any vaccination (RR = 1.5; 95% CI 1.0 to 2.3).

**Table 3:** Rate ratio of sudden unexpected deaths in infants of age 31–729 days by risk period (following any vaccination) and dose, Italy 1999–2004.

	Risk period: 0–1 days		Risk period: 0–7 days		Risk period: 0–14 days	
	N	RR adj^1^ (95% CI)	N	RR adj^1^ (95% CI)	N	RR adj^1^ (95% CI)
**All doses**	8	1.2 (0.4–2.1)	34	1.3 (0.9–1.9)	52	1.1 (0.8–1.5)
**1^st^ dose**	5	1.2 (0.4–2.5)	24	1.5 (1.0–2.3)	34	1.2 (0.8–1.6)
**2^nd^ – 3^rd^ dose**	3	1.2 (0.3–3.0)	10	1.0 (0.4–1.9)	18	1.0 (0.6–1.6)

N: Number of deaths; RR adj: adjusted Rate Ratio; CI: Confidence Interval.

1 RRs are estimated according to the self controlled case-series method for censoring, perturbed or curtailed post-event exposures [19] and adjusted by age group (31–80; 81–100; 101–120; 121–180; 181–360; 361–729).

Similar estimates for the exposure to any vaccines were obtained when the Poisson regression model was applied (Table 4). With regard to the three main groups of vaccines (“Hexavalent products”, “Other concomitant administration of six antigens”, and “Any other administration of antigens”) the estimates of the RRs were slightly greater than one for the categories of “Hexavalent products” and “Other concomitant administration of six antigens”. Specifically, the RRs for the “Hexavalent products” were 1.5 (95% CI 0.6 to 4.2), 2.0 (95% CI 1.2 to 3.5) and 1.5 (95% CI 0.9 to 2.4), respectively for the risk periods 0–1, 0–7 and 0–14. The risk estimates for the “Other concomitant administration of six antigens” were largely overlapping with those of “Hexavalent products”. The RRs for “Other vaccine administration” were lower than 1 for any of the three periods at risk.

**Table 4:** Rate ratio of sudden unexpected deaths in infants of age 31–729 days by risk period and type of vaccine, Italy 1999–2004.

Vaccine groups	Risk period: 0–1 days			Risk period: 0–7 days			Risk period: 0–14 days		
	N	P-d	RR adj^1^ (95% CI)	N	P-d	RR adj^1^ (95% CI)	N	P-d	RR adj^1^ (95% CI)
**Any vaccine**	**8**	**864**	**1.1 (0.5–2.4)**	**34**	**3355**	**1.4 (0.9–2.1)**	**52**	**6104**	**1.1 (0.8–1.6)**
**All concomitant administration of six antigens**	**7**	**593**	**1.5 (0.7–3.5)**	**30**	**2276**	**1.8 (1.1–2.8)**	**44**	**4112**	**1.5 (1.0–2.2)**
Hexavalent products^2^	4	322	1.5 (0.6–4.2)	18	1231	2.0 (1.2–3.5)	25	2228	1.5 (0.9–2.4)
Hexavac	1	160	0.7 (0.1–5.5)	12	599	2.8 (1.4–5.3)	13	1075	1.6 (0.8–3.1)
Infanrix hexa	3	160	2.3 (0.8–7.7)	6	624	1.4 (0.6–3.1)	12	1138	1.5 (0.8–2.7)
Other concomitant administration of six antigens	3	271	1.4 (0.4–4.8)	12	1045	1.6 (0.8–3.0)	19	1884	1.4 (0.8–2.3)
**Others**	**1**	**271**	**0.5 (0.1–3.4)**	**4**	**1079**	**0.5 (0.2–1.4)**	**8**	**1978**	**0.6 (0.3–1.1)**
**Control period**	**192**	**29875**	**1**	**192**	**29875**	**1**	**192**	**29875**	**1**

N: Number of deaths; P-d: Person-days at risk; RR adj: adjusted Rate Ratio; CI: Confidence Interval.

1 RRs are estimated by the Poisson regression model and adjusted by age group (31–80; 81–100; 101–120; 121–180; 181–360; 361–729).

2 The information of the brand name of the hexavalent product was missing for 1 infant (the event occurred in the control period).

The RR estimates of the two “Hexavalent products” (Hexavac and Infanrix hexa) differed according to the time window at risk considered in the analysis. Among subjects immunised with Hexavac, one death occurred in the day of vaccination or in the following day (RR = 0.7; 95% CI 0.1 to 5.5); 12 deaths in the risk period 0–7 (RR = 2.8; 95% CI 1.4 to 5.3); and 13 deaths (RR = 1.6; 95% CI 0.8 to 3.1) for the risk period 0–14. Among infants who received Infanrix hexa the RR ranged from 2.3 (95% CI 0.8 to 7.7) for the first 2 days, to 1.4 (95% CI 0.6 to 3.1) for the first week, to 1.5 (95% CI 0.8 to 2.7) for the two week risk period.

The analysis by dose for the combination of six antigens indicated that the increase in the rate ratio was confined to the first dose: the rate ratio for the two “Hexavalent products” was 2.2 (95% CI 1.1 to 4.4) in association with the first dose, and 1.0 (95% CI 0.5 to 2.1) for the second and third dose combined (Table 5). A similar pattern was observed for the two hexavalent products.

**Table 5:** Rate ratio of sudden unexpected deaths in infants of age 31–729 days for the risk period 0–14 days following vaccination with a combination of six antigens by dose, Italy 1999–2004.

	First dose			Second and third dose		
	N	P-d	RR adj^1^ (95% CI)	N	P-d	RR adj^1^ (95% CI)
**Any administration of six antigens**	**30**	**2457**	**1.9 (1.0–3.4)**	**14**	**1655**	**1.2 (0.7–2.1)**
**Hexavalent products** 2	**18**	**1263**	**2.2 (1.1–4.4)**	**7**	**965**	**1.0 (0.5–2.1)**
Hexavac	10	580	2.7 (1.1–6.9)	3	480	0.8 (0.3–2.6)
Infanrix hexa	8	668	1.9 (0.8–4.2)	4	485	1.1 (0.5–2.9)
**Other concomitant administration of six antigens**	**12**	**1194**	**1.6 (0.8–3.2)**	**7**	**690**	**1.4 (0.6–3.0)**
**Control period**	**192**	**29875**	**1**	**192**	**29875**	**1**

N: Number of deaths; P-d: Person-days at risk; RR adj: adjusted Rate Ratio; CI: Confidence Interval.

1 RRs are estimated by the Poisson regression model and adjusted by age group (31–80; 81–100; 101–120; 121–180; 181–360; 361–729 for the first dose; 31–180; 181–360; 361–729 for the second-third dose).

2 The information on the brand name of the hexavalent product was missing for 1 infant (the event occurred in the control period).

Three deaths occurred during the risk period 0–14 days after the administration of the third dose: 1 day after the administration of Hexavac (male, age 346 days); 7 days after the concomitant administration of five antigens (male, age 385 days) and 7 days after Infanrix hexa concomitantly administered with the first dose of pneumococcal vaccine (female, age 428 days). A further event occurred 12 days after the first dose of MMR (male, age 494 days).

## Discussion

Our study was motivated by the safety concern that was raised in Germany with regard to Hexavac [13] and by the magnitude of the population exposed to the same vaccine in Italy. In Germany between November 2000 and June 2003, three deaths had occurred in toddlers in the second year of life within the 48 hours following the administration of the fourth dose, out of around 1.3 million doses. The observed vs expected ratio gave an SMR (Standardised Mortality Ratio) of around 23. The signal was restricted to the fourth dose, whereas no signal was reported for the three doses administered in the first year of life.

The findings of our study do not reproduce the signal. The rate ratio observed in the Italian study, for any vaccine and for any risk period, even when greater than 1, were almost an order of magnitude lower than the SMR estimated in the Germany. Statistically significant RRs were observed for hexavalent products and in particular for Hexavac, but only in the first seven days following vaccination. Moreover differently from the signal in Germany, the highest RR estimate in our data is limited to the first vaccine dose, at an age when the incidence of SUD is also the highest.

The incidence of SUD decreases around the age corresponding to the administration of the first dose. As a consequence, the comparison between risk periods (days closer to the vaccine shot) and control periods (more distant days) is influenced by the decreasing trend of the basal rates of SUD. Thus, our RRs may be at least partly affected by the residual uncontrolled confounding effect of age.

With regard to the hexavalent products, the difference in the risk estimates between Hexavac and Infanrix hexa is due to the uneven distribution of events in the two weeks following vaccination, whereas the total number of cases and person days is similar: 13 cases and 1075 person days for Hexavac and 12 cases and 1138 person days for Infanrix hexa. For “Other concomitant administration of six antigens” the rate ratio estimates in the risk period 0–14 days are also similar to the estimates of the two hexavalent products. For “Other vaccine administration” the rate ratio estimates are slightly lower than 1 over any of the risk periods. However, it should be considered that this category concerns a heterogeneous groups of vaccines and a small number of events.

The results of a preliminary study relevant to the years 1999–2003 were made available and discussed at the CHMP-EMA in April 2005. After reviewing the overall available evidence the CHMP-EMA concluded that the benefit-risk balance for Hexavac remained positive and no further regulatory actions were considered to be necessary. In the same year, however, a potential risk associated with a decreased immunogenicity of the hepatitis B component of Hexavac was identified, and the suspension of the marketing authorisation of this vaccine was recommended in Europe [24]. Almost the entire population of Italian infants immunised with Hexavac was consequently included in the present study.

The main strength of our findings is that the study was carried out on the entire Italian population: around 95% of 3.2 million newborns over a six year study period were immunised in the first two years of life. During the period 2001–2004 around 4.5 million doses of Hexavac and Infanrix hexa, equally shared between the two products (and presumably between the three doses), were administered to Italian infants.

Several potential limitations should also be discussed. The mortality rate of SUD was extremely low even after reviewing the individual causes of death to enhance the sensitivity of case detection: around 17 per 100,000 newborns in the first year of life (corresponding to 538 eligible subjects), and 2 per 100,000 infants in the second year (corresponding to 67 eligible subjects).

The identification of SUD cases based on death certificates and not on autopsy referrals could be a limitation of the study as it may have led to the inclusion of subjects with a defined cause of death (not reported in the certificate). Also the revision of the death certificates did not allow to estimate the proportion of events in which the reported causes of death were based on the autopsy findings. Clearly, had the autopsy been conducted on all potential cases of SUD, further cases would have been excluded and the estimate of the mortality rate would have been even lower. The inclusion criteria inevitably required to be adapted to the retrospective design. In this respect, our study subjects differed from the case definition agreed by the Brighton collaboration, which was specifically developed for unexplained sudden death as an adverse event following immunisation [25]. However, the study population may be considered more representative of the events reported to the pharmacovigilance systems, given that spontaneous reporting does not require the autopsy confirmation.

Fifty-one of the 244 immunised cases (21%) were in their second year of life, and 42 (17%) had received the third dose. Thus, in the second year of life, or around the age when the third dose is administered in Italy (11–12 months), the statistical power of the study was probably low to rule out a small increase in the risk for any specific product. Nevertheless, the fact that, despite a broader definition of SUD, only two cases occurred in temporal relation with the third dose (1 and 7 days respectively after Hexavac, and Infanrix hexa in association with a pneumococcal vaccine, had been administered) in around 1.5 million infants who received the third dose of these products, represents in itself an information on the safety of vaccines.

The retrieval of immunisation status is unlikely to have biased our estimates. Vaccination history was ascertained on official records after the inclusion of cases. The likelihood that we may have missed vaccination histories of some cases may have reduced the power of the study. However, the 95% vaccination coverage observed in the general population is not directly relevant to our study since we only considered a subgroup of children who died at early age (the median age of the 604 eligible subjects was 107 days). For instance, 218 subjects had died between 31 and 80 days, when the likelihood of being vaccinated in the corresponding general population is negligible (given that the first dose is recommended around 90 days).

The fact that the proportion of vaccinated infants reached a maximum of around 75%, which is still 20% lower than the general population coverage, is not unexpected. For example, in a study carried out in the UK, out of 363 children, an interview was conducted for 325 (90%), immunisation details were available for 303 (93%), and the immunisation programme had begun for 149 (49%). These immunised children represented 41% of the eligible population, even though an accelerated schedule (at 2, 3, and 4 months) had been introduced [26].

A lower immunisation rate among SUD cases than among controls was observed in most of the studies conducted to date [3], [5], [6], [10], [11], [26], and an explanation based on the “healthy vaccinee effect” has been proposed [27]. If a child is affected by a condition associated both to the occurrence of SUD and to the avoidance or postponing of vaccination, the comparisons between immunised and not immunised subjects (carried out either in cohort or case-control studies) would be confounded and RRs underestimated. Study design in which only cases are enrolled have been proposed to address the problem of confounding related to the avoidance of vaccination [15]–[19].

A final issue concerns the potential implications of the study findings on the benefit-risk profile of hexavalent vaccines. The acceptance of even weak and uncertain associations with severe events is largely influenced by the incidence of the diseases targeted by the vaccines, which modifies the perception of the expected benefits. Effective vaccination campaigns, which succeed in eliminating the corresponding diseases, may result in considering unacceptable even extremely rare adverse effects. An indirect evidence of the potential benefits associated with the vaccination coverage is provided by the outbreak of severe diseases which reappears a consequence of a decrease in the immunisation coverage. The recent outbreak of a poliomielitis epidemics in Tajikistan, with hundreds of cases and several deaths, is only the last in a series of outbreaks of otherwise entirely preventable diseases [28]. These episodes provide further evidence of the largely positive benefit-risk profile of extensive immunisation programmes.

In conclusion, our findings do not confirm the signal raised in Germany of a 23 fold increase in the risk of SUD in association with the administration of a hexavalent vaccine. Differently from the German signal, where the increased risk concerned the forth dose (infants in the second year of life), in our study only the first dose, which is administered when the incidence of SUD is greater, appears to carry a smaller, though statistically significant, increase in the risk of SUD. The residual uncontrolled confounding effect of age may partly explain this finding. Given the extremely low mortality rate for SUD in Italy, the power of the study was too limited to rule out a small increase in the risk of death in association with any specific vaccine either during the second year of life or following the third vaccine dose. At present, even though many studies investigated the potential association between vaccinations and the occurrence of SUD, only limited evidence is relevant to the role of hexavalent vaccines. Further studies conducted on similar populations may contribute additional evidence and allow a meta-analysis to obtain more precise estimates.

Nevertheless, our findings are globally reassuring, when considering that around 4.5 million doses of hexavalent products were administered in Italy during the study period. They are also coherent with the limited number of SUDs reported to the Italian pharmacovigilance system after the conclusion of this study. During the period 2005–2009, three deaths were reported within two weeks following the administration of a hexavalent product, out of around 2.5 million vaccinated infants and 7.5 million doses.
